# Ku80 is highly expressed in lung adenocarcinoma and promotes cisplatin resistance

**DOI:** 10.1186/1756-9966-31-99

**Published:** 2012-11-27

**Authors:** Qingshan Ma, Ping Li, Minyu Xu, Jinzhi Yin, Zhenzhong Su, Wei Li, Jie Zhang

**Affiliations:** 1 Department of Respiratory Medicine, The Second Affiliated Hospital of Jilin University, Changchun, China; 2 Department of Pediatrics, The First Affiliated Hospital of Jilin University, Changchun, China

**Keywords:** Ku80, Lung cancer, Chemotherapy, siRNA, Cisplatin

## Abstract

**Background:**

Ku80 is crucially implicated in DNA repair, apoptosis, and chemoresistance. In this study, we aimed to assess the expression of Ku80 in clinical lung adenocarcinoma specimens, and investigate its role in the regulation of cisplatin sensitivity in cisplatin resistant human lung adenocarcinoma cells A549/DDP.

**Methods:**

Tumor specimens and medical records of 106 patients with operable lung adenocarcinoma were obtained from 1998 to 2003. Ku80 mRNA and protein levels of the tumor samples, cultured human lung adenocarcinoma cells A549 cells and their cisplatin resistant variant A549/DDP cells were examined by reverse transcription PCR and western blot analysis. Ku80-specific siRNA or control scramble siRNA was transfected into A549/DDP cells, then cell sensitivity to cisplatin was examined by 3-(4,5-dimethylthia-zol-2-yl)-2,5-diphenyltetrazolium bromide assay and apoptosis was assessed by flow cytometric analysis. In addition, the levels of cleaved caspase-3 and cleaved PARP in the treated cells were detected by western blot analysis.

**Results:**

Total 83.3% (20/24) cisplatin-resistant tumors had high Ku80 expression, while 8.3% (4/48) cisplatin-sensitive tumors had high Ku80 expression (p < 0.01). Univariate analysis indicated that overall survival and progression-free survival were significantly better in lung adenocarcinoma patients with low vs. high Ku80 expression level (p < 0.01). Ku80 mRNA and protein expression levels were significantly increased in A549/DDP cells compared to parental A549 cells. siRNA mediated knockdown of Ku80 resensitized A549/DDP cells to cisplatin-induced apoptosis.

**Conclusions:**

Ku80 expression level could predict the outcome and the sensitivity to cisplatin-based chemotherapy in patients with lung adenocarcima. Ku80-siRNA could be utilized as a therapeutic strategy to resensitize nonresponders to cisplatin.

## Background

Lung cancer is the leading cause of cancer death in males and the second-leading cause of cancer deaths in females worldwide [1]. In the past decades, lung adenocarcinoma, one histological subtype of non-small cell lung cancer (NSCLC), has become the most common histologic type among all lung cancers diagnosed [2]. Platinum based combination chemotherapy is the standard chemotherapy for NSCLC, and cisplatin is widely used for the treatment of lung cancer [3]. However, individuals respond to chemotherapy differently and the efficacy of cisplatin treatment is often impaired by the emergence of resistance to this drug [4]. Therefore, elucidating the mechanism underlying the development of chemoresistance would promote our understanding of lung cancer progression and treatment failure.

The heterodimeric Ku antigen, which acts as a molecular detector of DNA double strands, consists of two subunits of 70 kDa (Ku70) and 80 kDa (Ku80 or Ku86) and activate DNA protein kinase (DNA-PK) by binding directly to free DNA termini in a non-sequence-specific manner [5,6]. Expression of Ku was shown to be upregulated in human aggressive breast cancer, lung cancer and bladder cancer [7-10]. Moreover, Ku is involved in the resistance of ovarian cancer and leukemic cells to cisplatin [11-13]. However, little is known about the expression of Ku80 and its role in cisplatin resistance in human lung adenocarcinoma. Therefore, in this study, we assessed the expression of Ku80 in lung adenocarcinoma specimens, and found that Ku80 was markedly overexpressed in primary human lung adenocarcinoma and high Ku80 expression was associated with poor clinical outcomes and resistance to cisplatin-based chemotherapy. siRNA mediated knockdown of Ku80 enhanced the proapoptotic effects of chemotherapy on cisplatin-resistant lung adenocarcinoma cells A549/DDP.

## Materials and methods

### Patients and samples

Tumor samples from resection specimens were collected from patients with primary lung adenocarcinomas between January 1998 and July 2003, who underwent general thoracic surgery at the Second Hospital of Jilin University. The study was approved by the Ethics Committee of the Second Hospital of Jilin University (Changchun, China) and all patients gave informed consent. All excised tissues were frozen immediately in liquid nitrogen and then stored at −80 °C. Patient medical records were reviewed to obtain tumor staging, pathology, and survival information. The pathologic diagnosis of the resected tumors was based on the World Health Organization histological classification of tumors of the lung [14]. The post-operative disease stage was performed according to the International Union against Cancer's tumor-node-metastasis (TNM) classification [15]. All 106 patients underwent radical surgery. Patients with preoperative chemotherapy or radiotherapy treatment or with evidence of other malignancies were excluded. No patients received gene-targeted therapy during the follow-up period. Eighty-six patients received appropriate chemotherapy or radiotherapy as needed. Among them, 66 patients received more than three cycles of cisplatin-based chemotherapy. Platinum sensitivity, as a measure of treatment response, was defined as no disease progression or relapse during or within 6 months after chemotherapy [16]. The median clinical follow-up time was 38.5 months (range: 7–60 months). Overall survival was defined as the time from the diagnosis to death from any cause. Progression-free survival was defined as the time from the diagnosis to progressive disease, relapse or death from any cause, whichever occurred first. Cases lost to follow-up and deaths caused by conditions other than lung adenocarcinoma were regarded as censored data in the survival analysis.

### Immunohistochemistry

Paraffin-embedded tissue sections of primary lung adenocarcinoma and the adjacent normal lung tissues were used for immunohistochemical studies. Sections from paraffin-embedded tumors were incubated overnight with mouse anti-human Ku80 monoclonal antibody (Santa Cruz Biotechnology, Santa Cruz, CA) at 1:500 dilution, followed by incubation with goat anti-mouse secondary antibody (Pierce, Rockford, IL, USA). Immunohistochemical evaluation was performed by two pathologists without knowledge of the clinical and pathological characteristics of these patients. The average optical density and the average positive area ratio from five nonoverlapping, randomly selected fields per section with high-magnification (400×) were examined and counted. Average optical density reflected the positive intensity of area expressing Ku80 protein and equaled to average optical density of positive stained area. The positive area ratio reflected the scope of area with positive Ku80 expression and was calculated as (the total positive area per unit area/the total cells per unit area) × 100%. In the case of nuclear staining of Ku80, the percentage of positive cells was determined and divided into three groups: nuclear staining in less than 25% of cells (weak), nuclear staining in ≥25% of tumor cells and ≤50% of tumor cells (low) or nuclear staining in >50% of tumor cells (high).

### Cell lines and transfection

The human lung adenocarcinoma cell line A549 and its cisplatin-resistant variant A549/DDP were cultured as previously described [17]. Small interfering RNA (siRNA) sequences targeting human Ku80 and a non-target sequence were constructed by Genechem (Genechem, Shanghai, China). The Ku80 siRNA (si-Ku80) were designed with the following sequences as previously described [18,19]: sense 5'GGAUGGAGUUACUCUGAUUTT3', antisense 5'AAUCAGAGUAACUCCAUCCTT3'. The non-target siRNA (Scramble) sequences were as follows: sense 5'UUCUCCGAACGUGUCACGUTT3', antisense 5'ACGUGACACGUUCGGAGAATT3'. Transfection with siRNA was performed using LipofectAMINE 2000 (Invitrogen, Carlsbad, CA) in accordance with the manufacturer’s protocol. Briefly, A549/DDP cells were seeded into six-well plates at the density of 2 × 10^5^ cells/well, and the cells grew to 50-70% confluent in the next day. Then the cells were transfected with 100 pmol si-Ku80 or si-Scramble by using 10 μl LipofectAMINE 2000 (Invitrogen). For the 3-(4,5-dimethylthia-zol-2-yl)-2,5-diphenyltetrazolium bromide (MTT) assays and flow cytometry analysis, the transfected cells were treated with cisplatin for 24 h. The cells were harvested 48 h after transfection.

### Cell viability assay

The MTT staining kit (Sigma-Aldrich, St. Louis, MO) was used to determine cell viability. A549/DDP cells were plated into 96-well plates (1 × 10^4^/well) for 24 h and then treated with various concentrations of cisplatin for 24 h. Next the cells were treated with 0.5 g/l MTT solution for 4 h. The medium was removed, and 100 μl of dimethylsulphoxide was added to each well. The formazan dye crystals were solubilized for 15 min and the optical density was measured using a microplate reader (Bio-Rad, Richmond, CA) at a wavelength of 570 nm. All experiments were performed in triplicate.

### Flow cytometry analysis of apoptosis

After treatment for the defined time, A549/DDP cells were trypsinized and collected, washed, and stained using the Annexin-V-FITC Apoptosis Detection Kit (Beyotime, Shanghai, China). The samples were subjected to a FACScan flow cytometer (Becton Dickinson, Franklin Lakes, NJ). Annexin-V-FITC(−)/PI(−) was used to indicate cells that had survived, Annexin-V-FITC(+)/PI(−) was used to indicate cells in the early stage of apoptosis, and Annexin-V-FITC(+)/PI(+) was used to indicate cells in the late stage of apoptosis or necrosis.

### Reverse-transcriptase PCR analysis

Total RNA were isolated from cultured cells or tumor samples by using Trizol (Invitrogen, USA) according to the manufacturer’s instruction. Complementary DNA (cDNA) was synthesized by reverse transcription of 1 μg RNA samples with SuperScript pre-amplification system (Promega, Madison, MI). One tenth of the reverse transcribed RNA was used in PCR reaction. The primer sequences were as follows: GAPDH forward 5^′^ - GAAGGTGAAGGTCGGAGTC-3^′^ and reverse 5^′^- GAAGATGGTGATGGGATTTC^′^ (product 300 bp); Ku80 forward 5^′^-ACGATTTGGTACAGATGGCACT−3^′^ and reverse 5^′^-GCTCCTTGAAGACGCACAGTTT −3^′^ (product 497 bp). RT-PCR products were separated by electrophoresis on 1.5% agarose gel containing ethidium bromide.

### Western blot analysis

Total protein was isolated from culture cells or tumor samples and subjected to western blotting analysis as previously described [20]. Equal amounts of protein (40 μg) as determined by the Protein Assay Kit (Bio-Rad, Hercules, CA) was separated by 12% PAGE and transferred onto nitrocellulose membranes (Millipore, Bedford, MA). The membranes were blocked with 5% nonfat milk diluted in buffer (10 mM Tris–HCl, 100 mM NaCl and 0.1% Tween 20) for 1 h at room temperature. The membranes were then incubated with primary antibodies at 1: 1000 dilution for Ku80, cleaved-PARP, cleaved-Caspase 3, or β-actin (Abcam, MA, USA), followed by incubation with Horseradish peroxidase-conjugated secondary antibodies (Thermo, Waltham, USA) at 1: 2000 dilution for 1 h at room temperature. The protein bands were detected by an enhanced chemiluminescene kit (Pierce, Rockford, USA). Protein levels were quantified by densitometry using Quantity One software (Bio-Rad).

### Statistical analysis

The data were presented as mean ± standard deviation. All statistical analysis was performed using SPSS.17.0 software (SPSS, Chicago, IL, USA). The paired-samples Wilcoxon signed rank test was used to compare the expression of Ku80 between tumor and adjacent normal tissues. A 2-fold difference between control and test was considered the cut-off point to define high or low expression. Comparisons between treatments were made using one-way ANOVA for multiple group comparisons and differences between treatments were examined with a Tukey test. The correlation between Ku80 expression and clinic pathologic features was examined using the Pearson’s Chi-squared test. Overall survival and progression-free survival were calculated using the Kaplan–Meier method and log-rank tests. A 2-tailed P value of less than 0.05 was defined as statistical significance.

## Results

### Ku80 is overexpressed in lung adenocarcinoma tissues

**Figure 1:**
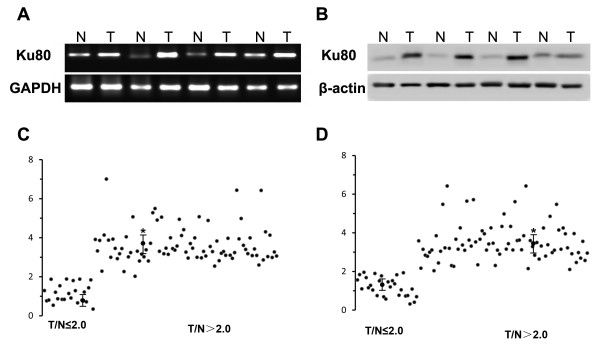
**Ku80 mRNA and protein expression in human lung adenocarcinoma tumor tissues.** (**A**) Ku80 mRNA level was detected by RT-PCR in tissue samples from lung adenocarcinoma tumor (T) and corresponding nontumorous (N) lung tissues. Shown were results from 4 representative paired-samples. (**B**) Quantitative data from A. (**C**) Ku80 protein level was detected by western blot in tissue samples from lung adenocarcinoma tumor (T) and corresponding nontumorous (N) lung tissues. Shown were results from 4 representative western paired-samples. (**D**) Quantitative data from C. *p < 0.01 compared to the normal tissues using Wilcoxon signed rank test.

**Figure 2:**
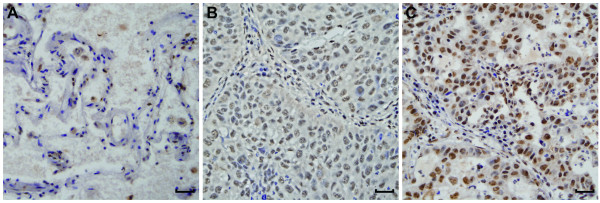
**Immunohistochemical staining of Ku80 in lung adenocarcinoma and adjacent nontumor lung tissues.** (**A**) Ku80 staining was weak in nontumorous lung tissue, (**B**) low level of expression of Ku80 in lung adenocarcinoma and (**C**) high level of expression of Ku80 in lung adenocarcinoma. Ku80 staining was brown and predominantly nuclear in the samples examined. Magnification × 400, scale bar 50 μm.

First we examined mRNA and protein expression of Ku80 in 106 pairs of snap-frozen lung adenocarcinoma and adjacent nonmalignant lung tissues. Representative data from four tumor and four non-tumor lung tissues were shown in Figure 1. As shown in Table 1, 73.8% (78/106) lung adenocarcinoma tissues showed high Ku80 mRNA expression (Figure 1A and C), and 78.3% (83/106) lung adenocarcinoma tissues showed high Ku80 protein expression (Figure 1B and D). By using a cutoff point of 2, we found that expression of Ku80 mRNA and protein was significantly reduced in lung adenocarcinoma vs. the non-tumor tissues (P = 0.006 and P = 0.005, respectively). A Spearman bivariate correlation showed a positive correlation (r = 0.97, P < 0.01) between the mRNA and protein levels of Ku80 (data not shown). Immunohistochemistry analysis demonstrated that Ku80 protein was expressed at low level in normal human lung tissues (Figure 2A) but at higher level in human adenocarcinoma tissues (Figure 2B and C) shown as nuclear brown-yellow granular staining.

**Table 1:** Association of Ku80 expression with clinical characteristics of 106 patients with lung adenocarcinoma

**Characteristics**	**patients (*n*** **= 106)**	***Ku80 mRNA level***		***P***	**Ku80 protein level**		***P***
		**Low (n = 28)**	**High (n = 78)**		**Low (* n *= 23)**	**High (*n * = 83)**	
Age at diagnosis of lung AC				0.202			
mean ± SD	58.33 ± 10.50	57.45 ± 9.96	60.79 ± 11.71				
Gender				0.371			0.151
Male	43(40.6)	9(32.1)	34(43.6)		6(26.1)	37(40.6)	
Female	63(59.4)	19(67.9)	44(56.4)		17(73.9)	46(55.4)	
Smoking status				0.238			0.13
Never	32(30.2)	11(39.3)	21(26.9)		10(43.5)	22(26.5)	
Former and current smokers	74(69.8)	17(60.7)	57(73.1)		13(56.5)	61(73.5)	
Tumor grade				0.062			0.114
Well differentiated	36(34.0)	15(53.6)	21(34.0)		12(52.2)	24(28.9)	
Moderately differentiated	32(30.2)	7(25.0)	32(30.2)		5(21.7)	27(32.5)	
Poorly differentiated	38(35.8)	6(21.4)	32(35.8)		6(26.1)	32(38.6)	
Lymph node metastasis				0.001			0.001
Positive	73(68.9)	11(39.3)	62(79.5)		6(26.1)	67(80.7)	
Negative	33(31.1)	17(60.7)	16(20.5)		17(73.9)	16(19.3)	
Disease stage				0.014			0.017
I	23(21.7)	10(35.7)	13(16.7)		10(43.5)	13(21.7)	
II	57(53.8)	13(46.4)	44(56.4)		9(39.1)	48(53.8)	
III	26(24.5)	5(17.9)	21(26.9)		4(17.4)	22(26.5)	

### Ku80 expression level is correlated with poor survival and resistance to cisplatin chemotherapy in lung adenocarcinoma patients

**Figure 3:**
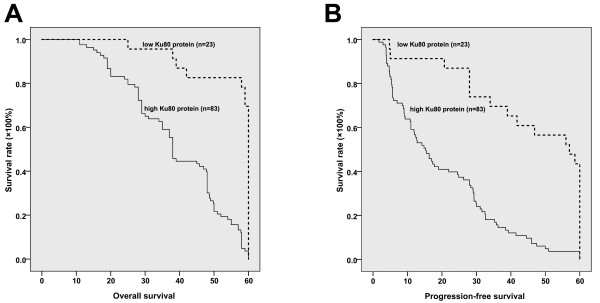
**Kaplan–Meier curve of overall survival of lung adenocarcinoma patients with low and high Ku80 expression.** (**A**) Kaplan–Meier analysis of tumor-specific overall survival in all lung adenocarcinoma patients according to Ku80 protein level. The 5-year survival probability was 94.4% for the patients with low Ku80 protein level (*n* = 23), and 79.8% for patients with high Ku80 protein level (*n* = 83). (**B**) Kaplan–Meier analysis of progression-free survival according to Ku80 protein level. The progression-free survival interval was 45.56 ± 3.85 (95% CI: 37.99-53.12) months for the patients with low Ku80 protein level (n = 23), and 20.18 ± 1.72 (95% CI: 16.81-23.54) for patients with high Ku80 protein level (n = 83).

We next addressed the relationship between Ku80 expression and clinicopathologic parameters of lung adenocarcinoma patients. As shown in Table 1, Ku80 overexpression showed significant correlations with lymph node metastasis status (P = 0.01) and TNM stage (P <0.05), but no correlation was noticed between Ku80 expression level and age, gender, smoking status or tumor grade. Analysis using the Kaplan–Meier method indicated that lung adenocarcinoma patients with high Ku80 level had a significantly shorter median overall survival compared to those with low Ku80 level (20.17 versus 57 months, P < 0.001 by the log-rank test; Figure 3A). Moreover, the progress-free interval was significantly higher in the low Ku80 level group than in the high Ku80 level group (P < 0.0001, Figure 3B). Taken together, these data demonstrate that Ku80 is overexpressed in primary lung adenocarcinoma compared with normal lung tissue, and high Ku80 level is associated with poor survival in lung adenocarcinoma patients.

In addition, as shown in Table 2, in this study 72 patients were treated with at least three cycles of cisplatin-based therapy, who were separated into cisplatin resistance group (n = 24) and cispaltin sensitivity group (n = 48) as defined previously [21]. Among these patients, 83.3% (20/24) cisplatin-resistant tumors showed high Ku80 expression level, while only 8.33% (4/48) cisplatin-sensitive tumors showed high Ku80 expression level. There was significant difference between the two groups (p < 0.01). These results suggest that Ku80 level is associated with the resistance to cisplatin-based chemotherapy in lung adenocarcinoma patients.

**Table 2:** The therapeutic status for patients with lung adenocarcinoma after surgery

**Stage**	**Number**	**Therapy after surgery**			
		**No therapy**	**Chemotherapy**		**Radiotherapy**
			**1-2 cycles**	**> 2 cycles**	
I	23	20	0	0	3
II	57	0	12	40	27
III	26	0	0	26	21
total	106	20	12	66	51

### Knockdown of Ku80 enhances cisplatin-induced apoptosis of cisplatin-resistant A549/DDP cells

**Figure 4:**
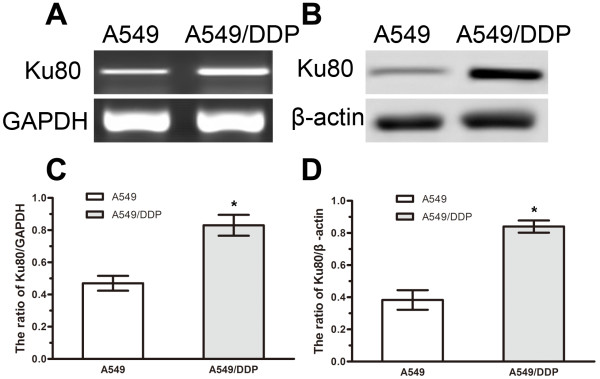
**Expression of Ku80 in A549 and A549/DDP cells.** (**A**) RT-PCR analysis of Ku80 mRNA in A549 and A549/DDP cells. (**B**) Western blot analysis of Ku80 protein in A549 and A549/DDP cells. (**C**) Quantification of Ku80 mRNA level relative to GAPDH. (**D**) Quantification of Ku80 protein level relative to β-actin. Data represented mean ± SD for three replicate experiments. *P < 0.05.

To characterize the role of Ku80 in the cisplatin resistance of lung adenocarcinoma, we examined Ku80 mRNA and protein expression in A549 and A549/DDP cells. Up-regulation of Ku80 at both mRNA and protein levels was detected in the cisplatin-resistant A549/DDP cells (Figure 4A and B), suggesting that increased expression of Ku80 promotes cisplatin resistance.

**Figure 5:**
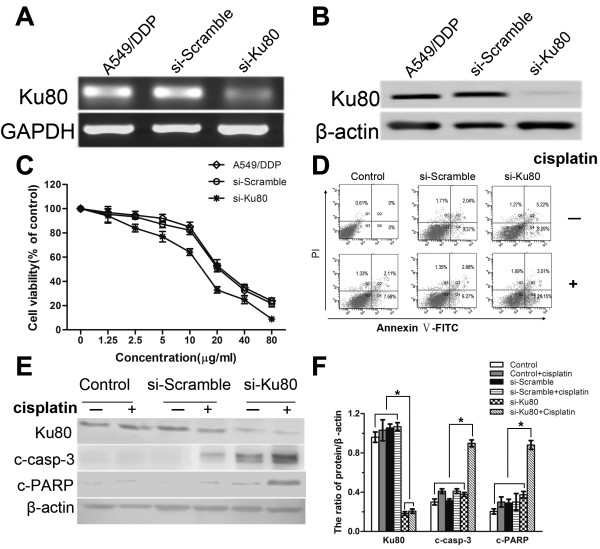
**Knockdown of Ku80 enhances cisplatin-induced growth inhibition and apoptosis in A549/DDP cells.** (**A**) RT-PCR analysis of Ku80 mRNA level in A549/DDP cells transfected with Ku80 siRNA (siKu80) or non-target sequence siRNA (Scramble). (**B**) Western blot analysis of Ku80 protein level in A549/DDP cells transfected with siKu80 or Scramble. (**C**) A549/DDP cells were transfected with siKu80 or Scramble and then treated with different concentrations of cisplatin for 24 h. Cell viability was determined by MTT assay. Data represented mean ± SD for three replicate experiments. (**D**) A549/DDP cells were transfected with siKu80 or Scramble and then treated with 6 μg/ml cisplatin for 24 h. The cells were collected and stained with Annexin-V-FITC and PI. Shown were representative images of three independent experiments. (**E**) A549/DDP cells were transfected with siKu80 or Scramble and then treated with 6 μg/ml cisplatin for 24 h. The cells were collected and subjected to western blot analysis for the detection of Ku80, cleaved caspase-3 and cleaved PARP levels. Shown were representative blots of three independent experiments. (**F**) Quantification of Ku80, cleaved caspase-3 and cleaved PARP levels as shown in (E). Data were presented as mean ± SD, n = 3. *P < 0.05.

To further confirm the effects of Ku80 on cisplatin sensitivity in human lung adenocarcinoma, we used siRNA to downregulate Ku80 expression in A549/DDP cells (Figure 5A and B). Cisplatin markedly increased the viability of si-Ku80 A549/DDP cells, whereas scramble-siRNA transfected cells were considerably less sensitive to cisplatin (Figure 5C).

The flow cytometry analysis showed that the apoptosis ratio was increased in siKu80-A549/DDP cells compared to scramble-siRNA transfected cells (24.16% vs. 12.15%, P < 0.05; Figure 5D). Furthermore, western blot analysis showed that si-Ku80 A549/DDP cells exhibited markedly increased activation of caspase-3 and cleavage of PARP in response to cisplatin, compared to scramble-siRNA transfected cells (Figure 5E and F). Collectively, these results suggest that Ku80 protects lung adenocarcinoma cells against cisplatin-induced apoptosis.

## Discussion

Platinum-based chemotherapies show promise in the treatment of lung cancer but their application has been limited by drug resistance [4]. Cisplatin, one classic platinum, has for many years been used as a systematic chemotherapeutic agent for the treatment of NSCLC [22]. Therefore, a better understanding of the mechanisms responsible for cisplatin resistance in lung cancer will improve the efficacy of cisplatin in clinical oncology.

In this study, we demonstrated that Ku80 is specifically up-regulated in lung adenocarcinoma compared to adjacent normal lung tissues. In addition, we found that increased Ku80 expression is associated with lymph node metastasis, TNM stage and tumor response to cisplatin-based adjuvant therapy, shorter overall and progression-free survival in patients with lung adenocarcinoma. The mechanism of action of cisplatin involves covalent binding to purine DNA bases, which primarily leads to cellular apoptosis. An increasing number of studies suggest that increased DNA repair capacity plays a critical role in cellular cisplatin resistance in many cancers including lung cancer [23,24]. Ku is known for its crucial role in DNA repair and may contribute to cisplatin resistance in lung adenocarcinoma. It has been shown that a rodent Ku80 knockout cell line exhibited hypersensitivity to cisplatin and reconstitution of human Ku80 in this cell line led to enhanced resistance to cisplatin [13].

Ku is implicated in numerous cellular processes, including telomere maintenance, regulation of specific gene transcription, regulation of heat shock-induced responses and apoptosis [25]. In this study, we demonstrated that siRNA mediated knockdown of Ku80 enhanced cisplatin sensitivity and promoted cisplatin-induced apoptosis as well as the activation of caspase-3 and PARP in cisplatin-resistant A549/DDP cells. Apoptotic pathways contribute to the cytotoxic action of cisplatin therapy [26]. Accordingly, the failure to undergo apoptosis in response to anti-cancer therapy may result in cancer resistance [27]. Caspase-3 plays a central role in the execution of the apoptotic program and is primarily responsible for the cleavage of PARP during cell death [28]. Cleaved caspase-3 indicates the activty of caspase-3, while PARP is a well-known substrate of caspase-3 and cleaved PARP indicates the extent of apoptosis. To further elucidate the possible mechanisms for Ku80 in cisplatin resistance, we examined the effects of Ku80-siRNA on cleaved caspase-3 and cleaved PARP. We observed that the levels of cleaved caspase-3 and cleaved PARP proteins were significantly increased in si-Ku80 transfected cells. Downregulation of Ku80, together with cisplatin treatment, might promote apoptosis by triggering caspases cascades in apoptotic pathways. However, further studies are needed to elucidate the mechanisms by which Ku80 downregulation promotes apoptosis of chemotherapy resistant cancer cells *in vivo* and *in vitro*. Li et al. reported that Ku80 inactivation resulted in the induction of the tumor suppressor protein p53, which may contribute to the inhibition of cell growth and induction of apoptosis [29]. Therefore, it will be interesting to examine the correlation between Ku80 expression and p53 mutation in lung adenocarcinoma patients.

In summary, our data suggest that Ku80 expression level could predict the outcome and the sensitivity to cisplatin-based chemotherapy in patients with lung adenocarcima. Ku80 knockdown increases the sensitivity of cisplatin resistant human lung adenocarcinoma cells to cisplatin *in vitro*. Therefore, Ku80 has the potential to serve as a biomarker for the prediction of cisplatin response and represent a promising target for the combination of cisplatin-based chemotherapy in patients with lung adenocarcinoma.

## Abbreviations

NSCLC: Non-small cell lung cancer; MTT: 3-(4,5-dimethylthia-zol-2-yl)-2,5- diphenyltetrazolium bromide; TNM: Tumor-node-metastasis.

## Competing interests

The authors declare that they have no competing interests.

## Authors’ contributions

QM and PL performed all the experiments and drafted the manuscript. MX and JY collected and provided the tissues. ZS and WL have contributed the data collection and interpretation. JZ oversaw the design of the study, was involved in the critically revised manuscript. All authors have read and approved the final version of the manuscript.
